# The genome sequences of lytic *Pseudomonas aeruginosa* bacteriophages BL1, BL2, and BL3 isolated from the environment

**DOI:** 10.1128/mra.01171-23

**Published:** 2024-03-01

**Authors:** Fahareen B. Mosharraf, Christopher Marpa, Karagen Rojas, Jeffrey Bernard, Austen Rowell, Lisa M. Bono

**Affiliations:** 1 Department of Biological Sciences, Texas Tech University, Lubbock, USA; Portland State University, USA

**Keywords:** bacteriophages, bacteriophage genetics, *Pseudomonas aeruginosa*, DNA sequencing

## Abstract

We isolated three environmental phages that infect *Pseudomonas aeruginosa* PAO1, an opportunistic pathogen, from Playa Lakes in Lubbock, TX. We report the genome sequences of isolated lytic bacteriophages BL1, BL2, and BL3. Sequence similarity analysis revealed that the viruses belonged to an unclassified species in the genus *Pbunavirus* within *Caudoviricetes*.

## ANNOUNCEMENT

*Pseudomonas aeruginosa* strain PAO1 is a gram-negative opportunistic pathogen causing infections in immunocompromised individuals (1–4). It is a top-priority pathogen due to antibiotic resistance and biofilm formation (5, 6). Here, we isolated phages that can infect *P. aeruginosa* PAO1 (generously provided by C. Wakeman at Texas Tech University) from Playa Lakes in Lubbock, Texas. We report three phages we isolated, sequenced, and characterized bioinformatically.

Samples which contained BL1 and BL2 bacteriophage were collected on 11 August 2022, and BL3 was collected on 13 October 2022. The GPS coordinates are 33.6165554,–101.8730933, 33.581,–101.883, and 33.5223491,–101.9016101, respectively. Each sample was obtained at a depth of 7–15 cm below water level. A 0.45-micron syringe filter was used to remove debris and sediment from the water sample. Subsequent filtration was done through a 0.22-micron syringe filter to remove bacteria. Briefly, 400 µL of exponentially growing *P. aeruginosa* PAO1 was inoculated with 200 µL of the filtered sample. The mixed culture was incubated at 37°C overnight. Upon centrifugation at 1,786 g for 30 min, the culture was filtered through 0.22-micron syringe filters to collect desired phages via the agar-overlay technique growing on the lawn of PAO1 strains (7). The phages were triple plaque purified. Phage genomes were extracted from phage lysates by Quantabio Extraction DNA Kit (Beverly, MA). DNA concentration and quantity were assessed by BioTek Take3 microvolume plate (Agilent, Santa Clara, CA). The extracted genomes were prepared for whole-genome sequencing using an Illumina DNA Prep Tagmentation Kit and were sequenced on the Illumina NextSeq 2000 (San Diego, CA) platform to generate 200-bp paired-end reads.

We trimmed raw Illumina reads and checked the quality with fastp v0.23.4 (8). We removed the host genome (NCBI accession no.: NC_002516.2) contaminants with Bowtie2 (9). Genome assembly was performed with SPAdes genome assembler v3.15.5 (10) using Illumina paired-end short reads. The coverage and depth were calculated with SAMtools v1.6 (11) and BEDTools v2.31.0 (12). Assembly statistics were generated using Quast v2.2.4 (13).  We used CheckV v1.0.1 (14) to assess the quality and completeness of our genome samples. Finally, NCBI BLASTn was used to perform the viral taxonomic characterization based on most similar sequences matched with NCBI nucleotide database, and genomes for all the bacteriophages were annotated with the Prokaryotic Virus Remote Homologous Groups database (Pharokka v1.3.2) (15, 16). To find the closest relative of our isolated phages available in the NCBI database, we used FastANI software v1.34 to determine the average nucleotide identity (Table 1), which runs a pairwise comparison of genomes to evaluate taxonomic relationships (17). We used default parameters for all the software. 

**TABLE 1:** Characterization of the reported bacteriophages

Parameter	Data for the isolated bacteriophages		
	BL1	BL2	BL3
Genome size (bp)	66,169	65,888	65,909
Total no. of reads generated per sample	493,096	460,044	465,503
Average coverage	1,490	1,396	1,412
CheckV completeness (%)	100%	100%	100%
CheckV quality	High	High	High
GC content (%)	54.62%	54.96%	55.58%
No. of coding sequence (CDs)	105	104	100
No. of hypothetical proteins	71	71	71
No. of tRNAs	0	0	0
Average nucleotide identity (ANI values in %)	96.34%	98.80%	98.37%
Closest related phage based on average nucleotide identity (GenBank accession no.)	*Pseudomonas* phage Epa6 MT108726	*Pseudomonas* phage misfit MT119367	*Pseudomonas* phage JJ01 ON324181
GenBank accession no.	OR733707	OR733708	OR733709
Bioproject accession no.	PRJNA1030388	PRJNA1030393	PRJNA1032425
SRA accession no.	SRP469478	SRP469327	SRP469477

Our SPAdes assembly metrics confirmed the existence of a lone contig (>5,000 bp) for each bacteriophage. Despite the results from CheckV, we were unable to define the start and stop sequence of four coding sequences, which is required by NCBI to be considered complete, so the genome sequences were considered partial. Based on sequence similarity, our analysis (Table 1) revealed that the viruses belonged to an unclassified species in the genus *Pbunavirus* within *Caudoviricetes*. CheckV identified no virulence genes or antibiotic resistance genes.

## Data Availability

Partial phage genomes were deposited in the GenBank, Bioproject, and NCBI Sequence Read Archive databases (SRA) under the accession numbers in Table 1.
